# Desmoplastic fibroma of the ilium: A case report with 4-month follow-up and literature review

**DOI:** 10.1097/MD.0000000000043503

**Published:** 2025-07-18

**Authors:** Lei Gao, Can Li, Wensong Zheng, Hong Yu

**Affiliations:** a Department of Medical Imaging, Hebei Medical University Third Hospital, Shijiazhuang, Hebei Province, China; b CT and MRI Center, Juancheng People’s Hospital, Heze, Shandong Province, China.

**Keywords:** bone tumor, case report, desmoid tumor, desmoplastic fibroma, ilium

## Abstract

**Rationale:**

Desmoplastic fibroma of the bone (DFB) is a rare and locally aggressive tumor that originates from the bone. We present a case of DFB involving the ilium that progressed over a 4-month follow-up period. To our knowledge, this is the first report detailing the short-term follow-up of DFB.

**Patient concerns:**

A 28-year-old male patient was admitted to our institution because of unexplained right iliac pain. Radiological assessment revealed osteolytic and expansive bone destruction in the right ilium along with linear hypointensity on T2-weighted imaging. After the 4-month follow-up, computed tomography images demonstrated an increase in lesion size.

**Diagnoses:**

The initial diagnosis was a fibrous tumor. Histopathological examination after the operation confirmed a diagnosis of DFB.

**Interventions and outcomes:**

The patient underwent extensive resection, reconstruction with bone cement, and internal fixation. No radiological evidence of tumor recurrence was observed 5 years after surgery.

**Lessons:**

This study provides the initial documentation of the short-term imaging progression characteristics of DFB in the iliac bone. Within the framework of the World Health Organization classification of bone tumors, DFB is categorized as an intermediate tumor based on its biological behavior. The case presented herein illustrates rapid progression of the tumor over a short period. Such findings underscore the paramount importance of achieving an accurate diagnostic evaluation through comprehensive imaging and the necessity of timely surgical intervention to effectively manage tumors and mitigate potential adverse outcomes.

## 1. Introduction

Desmoplastic fibroma of the bone (DFB) is a rare, locally aggressive, nonmetastatic primary bone tumor originally characterized by Jaffe^[1]^ in 1958. Similar to desmoid tumors found in soft tissues, DFB is histologically composed of abundant collagen fibers and spindle-shaped cells. This tumor can affect any part of the skeleton, with a predilection for the mandible and femurs.^[2]^ Its locally invasive behavior underscores the importance of an accurate preoperative diagnosis to guide the most appropriate surgical strategy. While long-term follow-up imaging studies of DFB (ranging from 3 to 9 years) have been previously reported,^[3,4]^ there is a paucity of data on short-term follow-up radiographic outcomes.

In this report, we describe a case of DFB with distinctive imaging features in the ilium that exhibited notable progression over a 4-month follow-up period. Our aim was to further understand the imaging manifestations of DFB and refine the accuracy of preoperative diagnosis by detailing imaging findings that have been pathologically verified.

## 2. Case data

### 2.1. General information and clinical examination

A 28-year-old male patient was admitted to our hospital on June 9, 2019, presenting with insidious, localized pain in the right iliac region, which had persisted for 6 months without radiation to other anatomical areas. The patient had no history of any trauma. Physical examination revealed exquisite tenderness and discomfort in the right iliac area, accompanied by antalgic gait. Laboratory analyses revealed elevated serum alkaline phosphatase (169 U/L; reference interval: 45–125 U/L), hyperuricemia (uric acid 674 μmol/L; reference interval: 208–428 μmol/L), and hypercalcemia (serum calcium 2.66 mmol/L; reference interval: 2.11–2.52 mmol/L). Other laboratory test results, including routine blood, urine, stool examinations, hepatic and renal function assessments, were all within the normal reference range.

### 2.2. Imaging presentation

**Figure 1.**
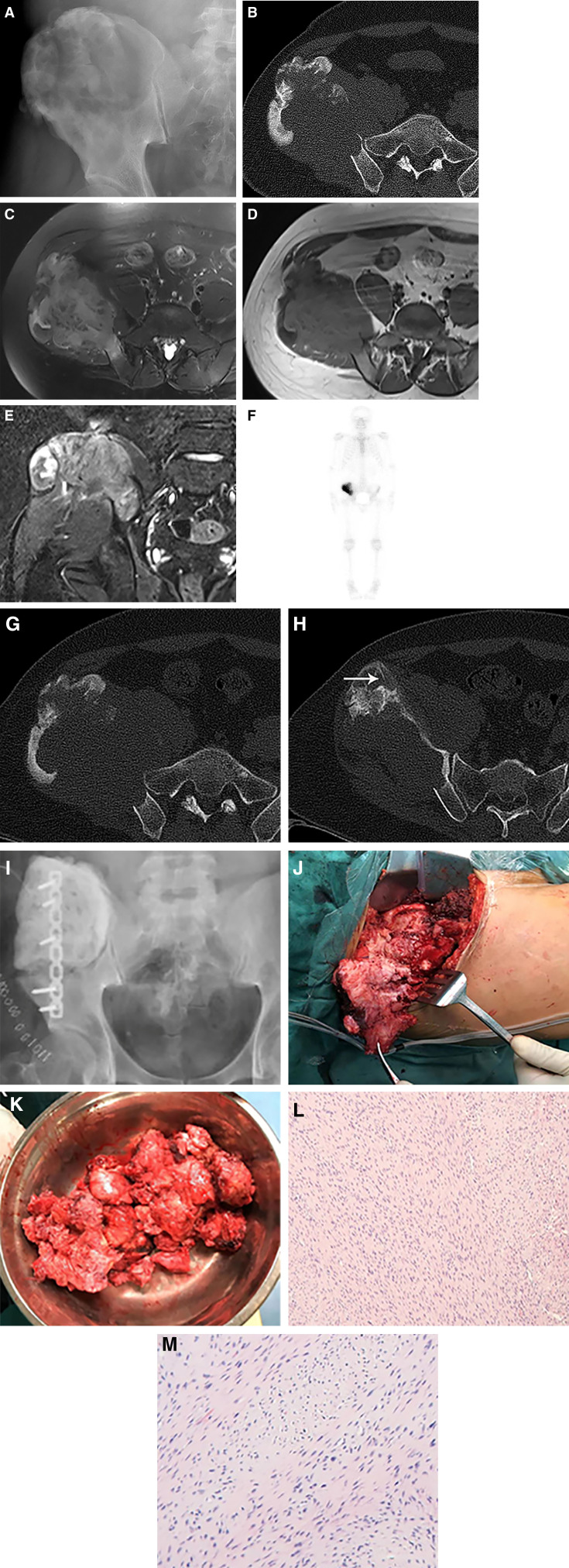
A 28-year-old man was admitted to our hospital due to the pain in right ilium without inducement persisting for the past 6 months. Digital radiography (A) showed an osteolytic destructive lesion of the right ilium. Transverse computed tomography(CT) (B) displayed an osteolytic and expansive bone destruction accompanied by high-density area at the edge and soft tissue mass in the right iliac bone. Transverse fat-suppressed T2-weighted imaging (C), T1-weighted imaging (D) and coronal fat-suppressed T2-weighted imaging (E) displayed a lesion rich in hypointense accompanied by linear areas of lower signal on T2-weighted imaging. Bone scintigram (F) showed heterogeneously increased tracer uptake in the right ilium. After a 4-month interval from initial admission, the transverse CT (G) showed increased extent of bone destruction and soft tissue mass in the right ilium accompanied by a reduced range of the high-density area at the edge. (H) Thick pseudo-trabeculation (arrow). (I) Postoperative image after tumor resection, bone cement reconstruction, and internal fixation. (J, K) The intraoperative images showed a tough fibrous tumor. (L, M) Abundant pink collagen fibers and spindle cells were found in the lesion [hematoxylin and eosin (HE), ×40 and × 100].

Digital radiography, computed tomography (CT), and magnetic resonance imaging (MRI) were conducted during the initial hospital admission. Digital radiography (Fig. 1A) revealed osteolytic bone destruction of the right ilium, accompanied by multiple high-density areas at the periphery of the lesion. CT (Fig. 1B) provided a more detailed visualization of the lesion, demonstrating extensive osteolytic bone destruction in the right ilium, localized loss of the bone cortex, and a massive soft tissue mass, with CT values ranging approximately 35 to 47 HU. The boundary of the bone destruction area lacked a sclerotic margin, whereas bone density increased near the iliac edge. MRI (Fig. 1C-E) revealed an isointense signal mass on T1-weighted imaging and hypointense signal on T2-weighted imaging (T2WI) with well-defined margins. ^99m^Tc-methylene diphosphonate bone scintigraphy (Fig. 1F) revealed heterogeneously increased tracer uptake in the right ilium.

After the imaging examination, the patient was advised to undergo surgical removal of the tumor. However, due to personal reasons, the patient opted for temporary conservative management. Four months later, the patient returned to our hospital for surgery and underwent a radiological examination. CT (Fig. 1G) demonstrated an increased extent of tumor-induced bone destruction, accompanied by a corresponding reduction in the high-density area at the iliac edge. Pseudo-trabeculation was also observed (Fig. 1H).

### 2.3. Outcome

**Figure 2.**
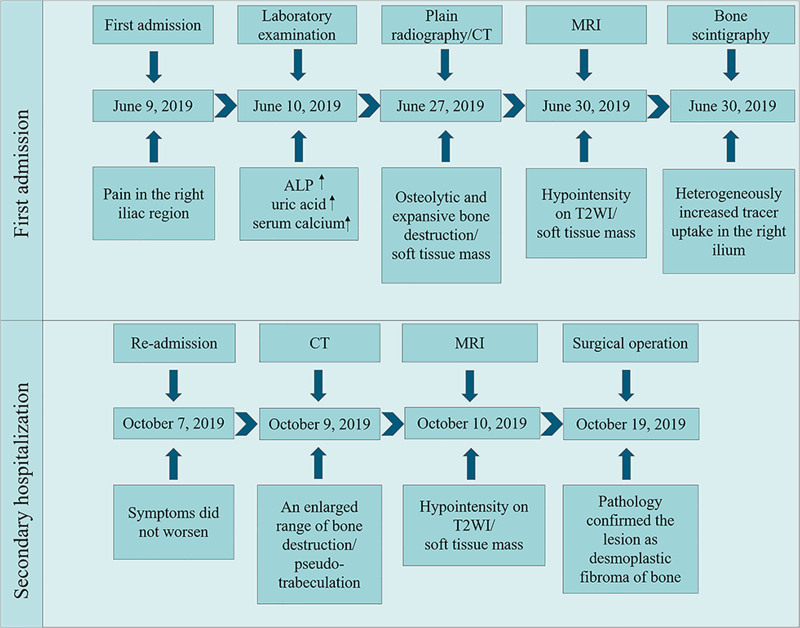
The timeline of the radiological imaging examination and diagnostic process of the patient in the present case. ALP = alkaline phosphatase, CT = computed tomography, MRI = magnetic resonance imaging.

Subsequently, the patient underwent pelvic tumor resection, followed by bone cement reconstruction and internal fixation (Fig. 1I). During surgery, a sizable tumor (approximately 20 × 18 × 6 cm) was identified in the ilium, demonstrating complete bone destruction. The tumor protruded into the extraosseous space, forming a firm mass (Fig. 1J, K). Histopathology revealed an abundance of eosinophilic collagen fibers and spindle-shaped fibroblasts within the lesion. Fibroblasts exhibited no significant atypia and mitotic figures were infrequent (Fig. 1L, M). The immunohistochemical results were as follows: Ki 67 (<10%), β-catenin (+), CD34 (−), SMA (−), desmin (−), and S-100 (−). The final pathological diagnosis was DFB. The timeline of examination and diagnosis after admission is shown in Figure 2.

In the present case, the patient underwent telephone follow-up, with no radiological evidence of recurrence detected after a 5-year postoperative follow-up. The patient was satisfied with surgical treatment.

## 3. Discussion

According to the World Health Organization Classification of Tumors of Soft Tissue and Bone,^[5]^ DFB is classified as an intermediate and locally aggressive tumor, implying that it may progress during follow-up, as demonstrated in this case involving the iliac wing. Although previous studies have reported long-term follow-up of DFB,^[3,4]^ there remains a lack of short-term follow-up imaging reports on this tumor entity. In this study, we present a case of DFB involving the ilium that demonstrated progression after a 4-month follow-up. To the best of our knowledge, this is the first report to detail the short-term progression of DFB. Imaging findings indicated that as a locally aggressive bone tumor, DFB can exhibit rapid progression, characterized by an expanded area of bone destruction and an increased soft tissue mass. These findings underscore the aggressive imaging features of DFB and provide compelling evidence for the critical importance of timely surgical intervention.

We conducted a comprehensive search of the online databases PubMed and Embase for reports on DFB published between January 1980 and October 2024, using the keywords “desmoplastic fibroma” or “desmoid tumor.” This search included 14 documented studies involving the ilium with a total of 21 cases. Among these, we obtained clinical information for 13 cases, and detailed radiological imaging findings were available for 11. The findings are summarized in Table 1.^[3,6–18]^

**Table 1:** Previously reported cases of desmoplastic fibroma of bone in ilium.

No.	Author/Year/number of cases	Gender	Age	Symptom	X-ray characteristics	CT characteristics	MRI characteristics	Treatment	Recurrence/follow-up time
1	Gebhardt et al^[6]^/1984/1	Male	28	/	Honeycombed appearance	Bone destruction with soft tissue mass	/	Resection allograft	No/5.5 years
2	Crim et al^[7]^/1989/1	Female	20	/	Pseudo-trabeculae lace-like appearance	/	/	En bloc resection	No/1 years
3	Inwards et al^[8]^/1991/2	/	/	/	/	/	/	Observation	Progressed slowly/11 years
4	Taconis et al^[9]^/1994/3	Male	20	/	Well-defined osteolytic lesion with ridges reactive bone formation	/	/	Resection	/
5	Nishida et al^[10]^/1995/1	Male	35	/	/	/	/	Curettage with graft	/
6	Stefanidis et al^[11]^/2011/1	Male	56	Lumbar pain	/	Lytic expansible lesion cortical breakthrough marginal sclerosis pseudo-trabeculation	T1WI: low signal STIR: high signal intensity contrast T1WI: heterogeneous increased signal	Curettage and intralesional bone grafting	No/6 years
7	Zhang et al^[12]^/2011/1	Female	49	/	Osteolytic bony lesion well-defined margin trabeculation	Lytic expansile lesion thin rim remnant trabeculae	/	Wide resection	/
8	Nedopil et al^[3]^/2013/1	Male	27	Increasing pain of right sacroiliac joint	/	Osteolytic bone destruction, intact sclerotic margin, pseudo-trabeculation, cortical destruction 3 years later	T1WI: low-signal intensity	Curettage and grafting	No/ 18 months
9	Rouchy et al^[13]^/2013/1	Male	15	Chronic left thigh pain	/	A well-defined bone lesion	FS-T2WI: Slightly lower signal with clear boundary		No/ 2 years
10	Evans et al^[14]^/2014/1	Female	29	Pain	/	/	/	No surgical treatment	/
11	Joshi et al^[15]^/2014/1	Female	39	Painless and gradually enlarged mass	Ill-defined expansile osteolytic lesion with soft tissue mass	Osteolytic lesion cortical destruction soft tissues	Ill-demarcated expansile mass T1WI: iso to low-signal intensity T2WI: heterogeneously increased intensity	Wide resection, plate fixation and autologous iliac bone grafting	No/6 years
12	Ma et al^[16]^/2017/1	Male	29	Minor pain	Osteolytic expansion lesion pseudo-trabeculation	Osteolytic expansion, cortical breakthrough, marginal sclerosis, pseudo-trabeculation	T1WI: low-signal intensity T2WI: high signal intensity	Curettage and grafting	No/ 3 months
13	Kinoshita et al^[17]^/2020/1	Female	21	Hip pain	Expansive osteolytic bone destruction with cortical destruction	Expansive osteolytic bone destruction, sclerotic margin, cortical destruction, slight pseudo-trabeculation	T1WI: low-signal intensity T2WI: low-signal intensity	Wide ilium resection	No/10 years
14	Zhang et al^[18]^/2022/5	Male	28	/	Map-like bone destruction thinned cortical bone “root whisker” trabecula or bone ridges	Expansile bone destruction, uneven density thinned and interrupted cortical bone invasion into soft tissue	T1WI: uneven equal or low-signal invasion of soft tissue	Tumor resection	/

CT = computed tomography, MRI = magnetic resonance imaging, T1WI = T1-weighted imaging, T2WI = T2-weighted imaging.

DFB is a rare, locally aggressive, and nonmetastatic primary bone tumor that accounts for approximately 0.1% of all bone tumors.^[5]^ It can occur at any age, particularly in individuals under 30 years of age, with males being more frequently affected.^[19,20]^ Of 14 cases involving the ilium, including the present case, there were 5 females and 9 males, with an age range of 15 to 56 years and a median age of 28. DFB can originate from the medullary cavity or bone cortex and affects various bones, most commonly the mandible,^[2]^ followed by the long bones (femur, radius, and tibia) and pelvic bones.^[5]^ In the literature reviewed, there were 4 cases involving the acetabulum, ischium, pubis, or sacrum simultaneously, suggesting that DFB may involve adjacent bones concurrently.^[6,11,16,17]^ Clinically, there is a lack of specificity in symptoms that are related to the tumor’s location and the presence of pressure on adjacent tissues. Pain accompanied by limited function is the most common clinical manifestation.

Radiographically, the common imaging manifestations include expansile lytic bone destruction. The bony cortex becomes thinner or disappears owing to invasion by soft tissue masses and extensive remodeling, indicating tumor aggressiveness, as demonstrated by the radiological findings in this case. The incidence of cortical destruction is approximately 23.1%.^[14]^ Five cases of ilium DFB recorded cortical bone destruction,^[3,11,15–17]^ with one case discovered during a 3-year follow-up,^[3]^ suggesting that these tumors can progress slowly. In this case, the range of the high-density area at the edge of the destruction area of the right ilium decreased, whereas the range of the mass increased, indicating accelerated tumor progression. Follow-up images obtained 4 months later confirmed this change.

DFB is typically characterized by nonsclerotic margins with a prevalence of 96%, as reported in the literature.^[21]^ In a comprehensive study involving 96 cases of DFB, sclerotic edges were identified in approximately 46% of the lesions.^[19]^ Among the 11 previously published retrospective cases with detailed radiological descriptions, 5 exhibited sclerotic margins.^[3,11,12,16,17]^ However, the lesion in our case did not demonstrate any evident sclerotic margins, suggesting the aggressive behavior of the lesion.

Furthermore, pseudo-trabeculation has been described in previous case reports. This phenomenon may present a range of appearances, from coarse linear strands to delicate lace-like patterns.^[7]^ It has been reported that the incidence of pseudo-trabeculation varies within 63% to 91%.^[7,19]^ Among 13 cases of iliac tumor, including the present case, pseudo-trabeculations were found in 10 cases (77%).^[3,6,7,9,11,12,16–18]^ The image manifestation of pseudo-trabeculation was described as “honeycombed appearance,” “pseudo-trabeculation,” “pseudo-trabeculae,” “root whisker trabecula,” and “bone ridges.” The formation of pseudo-trabeculation may be correlated with uneven bone destruction.^[7]^ Internal pseudo-trabeculation, which indicates trabecula-like bony septa within the tumor, facilitates the diagnosis of DFB. In DFB, numerous pseudo-trabeculations can engender multilocular and soapy-like structures. However, such structures should be distinguished from giant cell tumors (GCTs) in bones. Additional imaging features may also be conducive to diagnosis. For example, GCT on MRI is characterized by patches of low-signal intensity on T2WI, primarily attributable to hemosiderin deposition, whereas DFB is composed of fibrous tissue. Reports have indicated that the MRI signal of DFB is variable and nonspecific.^[21]^ However, the hypointensity on T2WI, which does not correspond to calcifications and is confirmed as areas of abundant collagen fibers that are pathologically paucicellular, may be beneficial in the diagnosis of DFB.^[21,22]^ The hypointense on T2WI corresponds to abundant collagen fibers, and the low-signal region has been histologically verified to be composed of compact fibroblasts. The case in our study exhibited a typical low signal in different regions, ranging from stripes to patches on T2WI, which corresponded to fibrous tissue on pathology, and fibrogenic tumors were correctly diagnosed preoperatively. In literature on DFB involving the ilium, 2 cases demonstrated low-signal intensity on T2WI.^[13,17]^ The extensive hypointensity on T2WI in our case is typical and can provide clues for an accurate diagnosis. It should be emphasized that focal low-signal intensity on T2WI, which does not correspond to calcifications, is mainly observed in fibrous dysplasia, nonossifying fibroma, and GCT. Other radiographic signs can assist in differentiating lesions for accurate diagnosis.

Previous studies have indicated that larger tumors may develop cystic degeneration, which often corresponds to mucoid degeneration.^[23]^ In a study assessing 8 cases of DFB, cystic changes were detected in 38% of the patients.^[23]^ Pathological examination confirmed the presence of fluid containing blood components within the cystic cavity. Relatively large DFB tumors are prone to cystic or myxoid degeneration.^[23,24]^ No cystic changes have been reported in iliac DFB, and this case did not exhibit any cystic changes either. Differentiating DFB from GCT can be challenging when hypointense lesions are observed on T2WI in masses that are accompanied by cystic changes. However, pathologically, GCT rarely exhibits fibrosis.

Therapeutic approaches include wide excision of the lesion, bone grafting, and internal fixation. Given the locally aggressive nature of DFB, extensive excision has been the primary approach in most of the reported cases. Total spondylectomy may be the most suitable therapeutic option for spinal tumors. Although these tumors do not exhibit metastatic behavior, local recurrence at the original site remains possible.^[25]^ Tumor recurrence is influenced by factors such as the presence of a soft tissue mass and the completeness of tumor resection. The scope of surgical intervention ranges from curettage and marginal resection to wide resection, with reported postoperative recurrence rates ranging from 15.4% to 72%.^[3,14]^ Prior literature on surgical curettage or resection of DFB in the ilium have demonstrated no recurrence throughout the follow-up period. In the present case, wide excision was performed, and no indications of tumor recurrence were revealed after a 5-year postoperative follow-up.

## 4. Conclusion

We present a case of ilium DFB that progressed and review previous reports on ilium DFB. The radiographic characteristics of osteolytic bone destruction accompanied by pseudo-trabeculation and hypointensity on T2WI are valuable for diagnosing DFB. A more comprehensive understanding of imaging characteristics would assist physicians in enhancing the precision of preoperative diagnosis, thereby ensuring that patients receive prompt and effective treatment.

## Author contributions

**Writing – original draft:** Lei Gao, Can Li.

**Writing – review & editing:** Lei Gao, Can Li, Wensong Zheng, Hong Yu.

**Visualization:** Wensong Zheng.

**Conceptualization:** Hong Yu.

**Supervision:** Hong Yu.

## References

[1] Jaffe HL. Tumors and Tumorous Conditions of the Bones and Joints. Lea & Febiger, 1958.

[2] Madakshira MG Bal A Verma RK. Desmoplastic fibroma of the mandible: a rare gnathic bone tumor with a review of the literature. Autops Case Rep. 2019;9:e2019091. 31641651 10.4322/acr.2019.091 PMC6771450

[3] Nedopil A Raab P Rudert M. Desmoplastic fibroma: a case report with three years of clinical and radiographic observation and review of the literature. Open Orthop J. 2013;8:40–6. 23459513 10.2174/1874325001307010040 PMC3583030

[4] Lu Y Lan W Wu Q. Desmoplastic fibroma in a child: a 9-year follow-up case report. BMC Musculoskelet Disord. 2024;25:306. 38643068 10.1186/s12891-024-07454-6 PMC11031886

[5] Board WCoTE. Soft Tissue and Bone Tumours:WHO Classification of Tumours. IARC Press, 2020.

[6] Gebhardt MC Campbell CJ Schiller AL Mankin HJ. Desmoplastic fibroma of bone. A report of eight cases and review of the literature. J Bone Joint Surg Am. 1984;67:732–47. 3997926

[7] Crim JR Gold RH Mirra JM Eckardt JJ Bassett LW. Desmoplastic fibroma of bone: radiographic analysis. Radiology. 1989;172:827–32. 2772196 10.1148/radiology.172.3.2772196

[8] Inwards C Unni K Beabout J Sim F. Desmoplastic fibroma of bone. Cancer. 1991;68:1978–83. 1913545 10.1002/1097-0142(19911101)68:9<1978::aid-cncr2820680922>3.0.co;2-h

[9] Taconis WK Schütte HE Heul RO. Desmoplastic fibroma of bone: a report of 18 cases. Skeletal Radiol. 1994;23:283–8. 8059254 10.1007/BF02412362

[10] Nishida J Tajima K Abe M. Desmoplastic fibroma aggressive curettage as a surgical alternative for treatment. Clin Orthop Relat Res. 1995:142–8. 7586818

[11] Stefanidis K Benakis S Tsatalou E Ouranos V Chondros D. Computed tomography and magnetic resonance imaging of desmoplastic fibroma with simultaneous manifestation in two unusual locations: a case report. J Med Case Rep. 2011;5:28. 21261947 10.1186/1752-1947-5-28 PMC3033348

[12] Zhang LL Yang HL Li XF Yuan J. Desmoplastic fibroma of ilium. Orthop Surg. 2011;3:216–8. 22009655 10.1111/j.1757-7861.2011.00136.x PMC6583631

[13] Couchy R Courvoisier A Wimsey S Bourgeois E Burroni B Griffet J. Desmoplastic fibroma of the ilium. Int J Surg Case Rep. 2013;4:875–8. 23973899 10.1016/j.ijscr.2013.06.016 PMC3785859

[14] Evans S Ramasamy A Jeys L Grimer R. Desmoplastic fibroma of bone: a rare bone tumour. J Bone Oncol. 2014;3:77–9. 26909301 10.1016/j.jbo.2014.08.001 PMC4723648

[15] Joshi N Heng W Huilin Y Weimin J. Giant desmoplastic fibroma in the right ilium: case report. J Med Erudite. 2014;2:1–6.

[16] Ma X Qiang S Liu T Cao M Lv S. Massive rare desmoplastic fibroma of the ilium and ischium in a young adult. Medicine (Baltimore). 2017;96:e8962. 29310397 10.1097/MD.0000000000008962 PMC5728798

[17] Kinoshita H Ishii T Kamoda H. Successful treatment of a massive desmoplastic fibroma of the ilium without surgery: a case report with long-term follow-up. Case Rep Orthop. 2020;2020:5380598. 32292618 10.1155/2020/5380598 PMC7148584

[18] Zhang ZZ Cao L Zhong ZW. Clinical and imaging features of desmoplastic fibroma of bone for correct diagnosis and differentiation. Curr Med Imaging. 2022;18:1093–8. 35410618 10.2174/1573405618666220411125408

[19] Frick MA Sundaram M Unni KK. Imaging findings in desmoplastic fibroma of bone: distinctive T2 characteristics. AJR Am J Roentgenol. 2005;184:1762–7. 15908527 10.2214/ajr.184.6.01841762

[20] Ishizaka T Susa M Sato C. Desmoplastic fibroma of bone arising in the cortex of the proximal femur. J Orthop Sci. 2021;26:306–10. 30097221 10.1016/j.jos.2018.07.011

[21] Vanhoenacker FM Hauben E Beuckeleer LHD Willemen D Marck EV Schepper AMD. Desmoplastic fibroma of bone: MRI features. Skeletal Radiol. 2000;29:71–175. 10.1007/s002560050589 10794556

[22] Gong L Liu W Ding Y Geng Y Sun X Huang X. Diagnosis and differential diagnosis of desmoplastic fibroblastoma by clinical, radiological, and histopathological analyses. Chin Med J (Engl). 2018;131:32–6. 29271377 10.4103/0366-6999.221274 PMC5754955

[23] Kim S Chung H Lee S Hong S Hwang J Kim M. Cystic changes in desmoplastic fibroma of bone: a new MRI finding. Clin Radiol. 2012;67:1170–4. 22656081 10.1016/j.crad.2012.04.009

[24] Okubo T Saito T Takagi T Suehara Y Kaneko K. Desmoplastic fibroma of the rib with cystic change: a case report and literature review. Skeletal Radiol. 2014;43:703–8. 24292164 10.1007/s00256-013-1772-7

[25] Kim O Kim S Kim JY. Desmoplastic fibroma of bone in a toe: radiographic and MRI findings. Korean J Radiol. 2013;14:963–7. 24265574 10.3348/kjr.2013.14.6.963 PMC3835646

